# Quantitative Trait Loci Mapping and Development of KASP Marker Smut Screening Assay Using High-Density Genetic Map and Bulked Segregant RNA Sequencing in Sugarcane (*Saccharum* spp.)

**DOI:** 10.3389/fpls.2021.796189

**Published:** 2022-01-05

**Authors:** Yijing Gao, Shan Zhou, Yuxin Huang, Baoqing Zhang, Yuhui Xu, Gemin Zhang, Prakash Lakshmanan, Rongzhong Yang, Hui Zhou, Dongliang Huang, Junxian Liu, Hongwei Tan, Weizhong He, Cuifang Yang, Weixing Duan

**Affiliations:** ^1^Guangxi Key Laboratory of Sugarcane Genetic Improvement, Key Laboratory of Sugarcane Biotechnology and Genetic Improvement (Guangxi), Ministry of Agriculture and Rural Affairs, Sugarcane Research Center, Sugarcane Research Institute, Guangxi Academy of Agricultural Sciences, Chinese Academy of Agricultural Sciences, Nanning, China; ^2^Adsen Biotechnology Co., Ltd., Urumchi, China; ^3^Interdisciplinary Research Center for Agriculture Green Development in Yangtze River Basin, College of Resources and Environment, Southwest University, Chongqing, China; ^4^Queensland Alliance for Agriculture and Food Innovation, University of Queensland, St Lucia, QLD, Australia; ^5^Guangxi Academy of Agricultural Sciences, Nanning, China

**Keywords:** sugarcane, smut, quantitative trait loci (QTL) mapping, bulked segregant RNA sequencing (BSR-seq), kompetitive allele-specific PCR (KASP) markers

## Abstract

Sugarcane is one of the most important industrial crops globally. It is the second largest source of bioethanol, and a major crop for biomass-derived electricity and sugar worldwide. Smut, caused by *Sporisorium scitamineum*, is a major sugarcane disease in many countries, and is managed by smut-resistant varieties. In China, smut remains the single largest constraint for sugarcane production, and consequently it impacts the value of sugarcane as an energy feedstock. Quantitative trait loci (QTLs) associated with smut resistance and linked diagnostic markers are valuable tools for smut resistance breeding. Here, we developed an F_1_ population (192 progeny) by crossing two sugarcane varieties with contrasting smut resistance and used for genome-wide single nucleotide polymorphism (SNP) discovery and mapping, using a high-throughput genotyping method called “specific locus amplified fragment sequencing (SLAF-seq) and bulked-segregant RNA sequencing (BSR-seq). SLAF-seq generated 148,500 polymorphic SNP markers. Using SNP and previously identified SSR markers, an integrated genetic map with an average 1.96 cM marker interval was produced. With this genetic map and smut resistance scores of the F_1_ individuals from four crop years, 21 major QTLs were mapped, with a phenotypic variance explanation (PVE) > 8.0%. Among them, 10 QTLs were stable (repeatable) with PVEs ranging from 8.0 to 81.7%. Further, four QTLs were detected based on BSR-seq analysis. aligning major QTLs with the genome of a sugarcane progenitor *Saccharum spontaneum*, six markers were found co-localized. Markers located in QTLs and functional annotation of BSR-seq-derived unigenes helped identify four disease resistance candidate genes located in major QTLs. 77 SNPs from major QTLs were then converted to Kompetitive Allele-Specific PCR (KASP) markers, of which five were highly significantly linked to smut resistance. The co-localized QTLs, candidate resistance genes, and KASP markers identified in this study provide practically useful tools for marker-assisted sugarcane smut resistance breeding.

## Introduction

Sugarcane (*Saccharum* spp. hybrids) is world’s largest sugar and the second largest bioethanol crop (FAOSTAT, 2020). Use of bioethanol as transportation fuel is steadily increasing globally, and, in 2019–2020 Brazil alone produced ∼33 billion liters of ethanol from sugarcane.^1^ In response to the growing sugar and biofuel demand, sugarcane production rose by about 45% in the last few decades globally, mostly through the expansion of cultivation in Brazil and India, the two largest sugarcane producers (FAOSTAT, 2020). Sugarcane is considered to be one of the most suitable renewable energy crops with potential for product diversification. Its industrial advantages include wide geography, currently grown in > 110 countries in tropics and sub-tropics, well-established breeding, production, processing and marketing systems, mostly rainfed, and more importantly it is already an established industrial-scale feedstock for electricity and ethanol. China is the third largest sugarcane producer in the world. In China, sugarcane is a major strategic crop and forms a component of expanding renewable energy portfolio underpinning government policies aimed at transitioning China to a carbon neutral country by 2050. It is grown in the Southern tropical and sub-tropical regions with ∼68% of cultivation located in Guangxi Province (Li, 2010).

Modern sugarcane cultivars are interspecific hybrids derived from crosses between *Saccharum officinarum* (2*n* = 80, *x* = 10), a species that accumulates unusually high concentrations of sucrose in their stalks, and *Saccharum spontaneum* (2*n* = 40–128, *x* = 8), a vigorous and widely adapted wild species with resistance to several sugarcane pests and diseases, tolerance to abiotic stresses and good ratoonability (D’Hont et al., 1996). These interspecific crosses have resulted in asymmetric chromosome transmission, producing varieties with different chromosome numbers ranging from 100 to 130 (Roach, 1969; D’Hont and Glaszmann, 2001). Due to its interspecific origin and higher order polyploidy, crosses between different sugarcane varieties produce aneuploid progeny (Roach, 1969; D’Hont and Glaszmann, 2001). Although the whole-genome sequence of *S. spontaneum* (Zhang et al., 2018) and a monoploid sequence of a commercial cultivar, R570 (Olivier et al., 2018), have been released, the large and complex genome structure of modern sugarcane hybrids makes genetic and genomic studies, including breeding of sugarcane very challenging.

This is reflected in the long history of molecular marker research, yet development of large-scale practically useful molecular markers for commercial breeding is only now becoming a significant research area, and that too restricted to a very few major sugarcane breeding programs worldwide (Aitken, 2021; Cursi et al., 2021; Ram et al., 2021; Wei et al., 2021).

Independent trait-specific sugarcane molecular genetic studies, however, have identified several disease resistance loci through quantitative trait locus (QTL) mapping and association mapping. For example, using a restriction fragment length polymorphism (RFLP) genetic map developed with 77 selfed progeny of cultivar R570 a major locus of sugarcane brown rust resistance, *Bru*1, was discovered (Daugrois et al., 1996). A genetic map containing 852 markers, including RFLPs, simple sequence repeats (SSRs), and amplified fragment length polymorphisms (AFLPs), constructed using 192 sugarcane F_1_ progeny was used to identify three resistance gene analogs (RGAs) which were used to generate markers that are significantly linked to brown rust resistance (McIntyre et al., 2005a) and sugarcane pachymetra root rot resistance QTLs (McIntyre et al., 2005b). Similarly, sugarcane brown rust resistance gene *Bru*2, a major QTL for sugarcane yellow spot disease resistance, and the sugarcane yellow spot resistance gene *Ryl*1 were also identified (Raboin et al., 2006; Aljanabi et al., 2007; Costet et al., 2012). Recently, using high-density genetic maps, Yang et al. (2018) located two new major QTLs for sugarcane brown rust resistance (Yang et al., 2017) and also identified molecular markers closely related to sugarcane orange rust resistance. In another study, You et al. (2019) used a single nucleotide polymorphism (SNP) array for genetic map construction and identified 18 QTLs controlling sugarcane yellow leaf virus resistance. Applying bulk-segregant analysis based on the sequencing (polyBSA-seq) strategy, three resistance- and one susceptibility-related candidate linkage markers for sugarcane leaf blight resistance were identified (Wang et al., 2021). Based on association mapping, 20 markers significantly associated with the four most important diseases in the Australian sugarcane industry, pachymetra root rot, leaf scald, Fiji leaf gall and smut, were obtained (Wei et al., 2006). Genome-wide association studies (GWAS) have discovered markers (DArT and AFLP) significantly associated with the sugarcane yellow leaf virus (SCYLV) (Debibakas et al., 2014). Association mapping method was also used based on 119 sugarcane genotypes fingerprinted for 944 SSR alleles, and four sugarcane red rot resistance markers were obtained (Singh et al., 2016). However, to date, *Bru1* PCR diagnostic markers for identifying brown rust resistant cultivars remains the only example of marker-assisted selection (MAS) in sugarcane (Costet et al., 2012; Glynn et al., 2013; Racedo et al., 2013; Li et al., 2016; Neuber et al., 2017). Thus, studies validating sugarcane DNA markers in breeding program or germplasm identification remain very limited.

Smut caused by *Sporisorium scitamineum*, initially reported in Natal in South Africa in 1877, has been one of the major sugarcane diseases worldwide (Mcmartin, 1945). In China the entire sugarcane production experiences yield losses caused by sugarcane smut, ranging from 10 to 50% depending on the year with serious economic consequences (Wei et al., 2012). Greater crop loss from smut occurs in low rainfall season. For example, the average cane yield in Guangxi province, which accounts for nearly 70% of sugarcane production in China, in 2020 was 61.5 ton ha-1 compared with 73.5 ton ha-1 in 2019, with increasing smut incidence accounting for most of the yield loss (Guangxi Sugar Association, 2020). Developing and utilizing resistant cultivars is the most efficient, economical and environment friendly approach for controlling smut disease (Croft, 2008). Hence, breeding for smut resistance is a key strategy to improve and stabilize the supply of sugarcane for sugar and lignocellulosic feedstock for energy production. Molecular breeding for sugarcane smut resistance so far focused on developing markers and identifying and cloning genes associated with smut resistance. Early in this work, much of the effort was directed to genetic mapping of smut resistance using AFLP markers and reported many markers with little effects (Raboin et al., 2001, 2003). Two markers linked to sugarcane smut resistance were obtained using bulked segregant analysis (BSA) (Xu and Chen, 2004; Gao et al., 2013). Association mapping using a panel of 154 Australian sugarcane clones of broad genetic base derived from diverse pedigree found 59% of the phenotypic variation in smut resistance ratings to be accounted for, by 11 markers (Wei et al., 2006). Other attempts to study smut resistance markers were mostly conducted with one or a few cultivars or elite clones, limiting the application of their findings (Esh and Nasr, 2014; Khan et al., 2017). Other research approaches reported include gene expression-based cloning of differentially expressed sugarcane genes (DEGs) (Thokoane and Rutherford, 2001) and NBS-LRR-type RGAs related to smut resistance, with the latter also used to study the molecular mechanism underpinning smut resistance (Que et al., 2008).

Most of the sugarcane smut research in the last two decades was aimed at developing markers for smut resistance, with a few reports on the discovery of QTLs for smut resistance (Raboin et al., 2001, 2003; Wei et al., 2006). The development of relatively low-cost large-scale genotyping and high-throughput sequencing technologies are now providing opportunities for more efficient molecular marker and gene discoveries, and genetic association studies. Considering the availability of genetic resistance for smut in the breeding population and the increasing use of high-throughput genotyping and marker discovery platforms, we hypothesize that developing a rapid and reliable screening assay based on molecular markers closely linked to smut resistance genomic loci would accelerate the development of smut-resistant sugarcane varieties to boost crop productivity. To this end, in the present study, we developed an F_1_ population derived from a cross between two hybrid sugarcane varieties, one smut resistant and the other smut susceptible, and used this for genetic mapping, and marker and candidate gene discovery research. In this next generation sequencing (NGS) era, sequencing-based technologies can provide novel strategies for genome-wide SNPs development and help construct a high-density genetic linkage map for high resolution QTL identification (Rehman et al., 2020). SNP markers can be called in many ways, including reduced-representation sequencing, re-sequencing and transcriptome sequencing. Reduced-representation sequencing has differentiated into different technologies, including specific-locus amplified fragment sequencing (SLAF-seq) (Sun et al., 2013), genotyping-by-sequencing (GBS) (Elshire et al., 2011), restriction site-associated DNA Sequencing (RAD-Seq) (Arnold et al., 2013), and 2b-restriction site-associated DNA sequencing (2b-RAD) (Wang et al., 2012), etc. SLAF is an effective and practical SNP discovery method for high diversity and large genome species, even without reference genome assemblies, which has been widely adopted in genotyping and genetic map construction (Wang et al., 2019; Zhang et al., 2019; Lyu et al., 2020; Song et al., 2020; Wei et al., 2020). In this study, we used SLAF-seq that allows locus-specific deep sequencing for genotyping accuracy, and cost reduction through reduced genomic representation scheme (Sun et al., 2013). The specific aims of our research reported here are: (i) SLAF-seq based integrated genetic map construction with SNPs and additional SSR markers from the biparental mapping population described above, (ii) identification of QTLs and SNP markers stably associated with smut resistance based on the integrated (SNP and SSR markers) genetic map, (iii) detection of QTLs and candidate gene mining for smut resistance via the bulked segregant RNA sequencing (BSR-seq) method, which was used for the first time in sugarcane, and (iv) development and evaluation of KASP markers assay with potential for screening large populations. For single point genotyping, kompetitive allele specific PCR (KASP) technology utilizes a unique form of competitive allele-specific PCR that enables highly accurate bi-allelic scoring of SNPs and InDels at specific loci across a wide range of genomic DNA samples, including those of complex genomes. It delivers high levels of assay robustness and accuracy with significant cost savings (Jatayev et al., 2017; Steele et al., 2018; Makhoul et al., 2020).

## Materials and Methods

### Plant Materials and DNA Extraction

The sugarcane mapping population used in this study included 192 F_1_ progeny from a cross between sugarcane varieties GT21 and ROC25, which were bred by the Sugarcane Research Institute of the Guangxi Academy of Agricultural Sciences (SRI-GXAAS) and the Taiwan Sugar Company, respectively, in China. GT21 is susceptible to smut disease caused by *S. scitamineum*, while ROC25 is resistant to smut (Gao et al., 2013). In addition, three standard control varieties, NCo376 (highly resistant), F134 (susceptible to *S. scitamineum* race 2, one of the major two races of the pathogen present in China, but resistant to race 1), and NCo310 (susceptible to race 1 but resistant to race 2), were included.

Genomic DNA was extracted from young leaves using the SDS method (Huang et al., 2010), quantified by spectrophotometry (260/280 nm) and quality was evaluated by agarose gel electrophoresis (1%). The DNA samples were diluted to 40 ng/μL in sterile deionized water and stored at –20°C.

### Smut Resistance Evaluation

The whole F_1_ mapping population, along with the two parental clones and the control varieties (NCo376, F134 and NCo310) were evaluated for smut resistance in four consecutive seasons from 2015 (year 1) to 2018 (year 4). All clones were artificially inoculated under greenhouse condition (22°85′N, 108°25′E).

The single-spore isolation of *S. scitamineum* collected from F134 was performed and the clones were inoculated following the protocol described previously (Chen et al., 2013). The “+” and “–” mating types of the sporidia of *S. scitamineum* race 2 were activated and cultured. The concentration of spores in the spore suspension was adjusted to 2 × 10^9^ spores/mL with sterile distilled water before inoculation, and the “+” and “–” mating type cultures were then mixed together at a ratio of 1:1. The test materials (nodal stem cuttings) were punctured four times around each bud with an insect needle, soaked in the spore suspension for 10 min and then incubated in the dark (28 ± 1°C) for 24 h. A total of 40 buds per clone were planted in a perforated plastic tray filled with sand and two replicates (trays) were maintained for each clone.

The incidence of smut whip emergence was recorded. The trial was screened for smut incidence every 7 days in the initial infection stage (first 3 months from smut inoculation) and then for every 15 days until the end of the experiment (6 months after inoculation). The smut-infected plants with emerging whips, once counted, were removed from the experiment immediately. For every experiment, the total number of experimental plants and the total number of smut-infected plants were recorded. Using the total number of infected plants, the smut incidence (%) was calculated. The smut response of clones was determined according to the disease incidence severity using a 1–9 rating scale (Que et al., 2006; Table 1).

**TABLE 1:** Smut resistant ratings of sugarcane varieties GT21 and ROC25 and the F_1_ mapping population derived from their crossing across Standard varieties NCo310, NCo376 and F134 were used as control.

Resistance rating	Disease incidence (%)	Clone resistance reaction
1	0–3	High resistance (HR)
2	4–6	Resistance (R)
3	7–9	Resistance (R)
4	10–12	Moderate resistance (MR)
5	13–25	Moderate susceptibility (MS)
6	26–35	Susceptibility (S)
7	36–50	Susceptibility (S)
8	51–75	High susceptibility (HS)
9	76–100	High susceptibility (HS)

$$\text{Incidence}\left(\%\right)=\frac{\mathrm{Number}\,\mathrm{of}\,d\,\text{ise}\,\mathrm{ased}\,\mathrm{plants}}{\text{To}\,\mathrm{tal}\,\mathrm{number}\,\mathrm{of}\,\mathrm{plants}}\times 100\%$$

### Specific-Locus Amplified Fragment Sequencing Library Construction and High-Throughput Sequencing

We followed SLAF-seq strategy and methodology as described by Sun et al. (2013), with slight modification. Briefly, genomic DNA was digested with HaeIII (New England Biolabs, NEB, United States) and a single-nucleotide (A) overhang was subsequently added to the digested fragments using the Klenow fragment (3′→5′exonuclease) (NEB) and dATP at 37°C. Then, Duplex tag-labeled sequencing adapters (PAGE-purified, Life Technologies, United States) were ligated to the A-tailed fragments using T4 DNA ligase. Polymerase chain reaction (PCR) was performed using the diluted restriction-ligation DNA samples, dNTPs, Q5 High-Fidelity DNA Polymerase, and PCR primers (forward primer: 5′-AATGATACGGCGACCACCGA-3′, reverse primer: 5′-CAAGCAGAAGACGGCATACG-3′) (PAGE-purified, Life Technologies). The PCR products were purified using Agencourt AMPure XP beads (Beckman Coulter, United Kingdom), pooled and they were separated by 2% agarose gel electrophoresis. Fragments that ranged from 364 to 444 base pairs in size (with indexes and adaptors) were separated and purified using a QIAquick gel extraction kit (Qiagen, Germany). The gel-purified products were diluted and used for paired-end sequencing (each end 125 bp) on an Illumina HiSeq 2500 system (Illumina, Inc.; San Diego, CA, United States). The curated data has been submitted to the CNGB sequence Archive (CNSA) of the China National GeneBank DataBase (CNGBdb; accession ID CNP0002008).

### Specific-Locus Amplified Fragment Sequencing Data Grouping and Genotyping

Genotyping and SLAF marker identification were performed as described previously (Sun et al., 2013). Briefly, low-quality reads (Q30 < 20) were filtered out, and all SLAF paired-end reads with clear index information were clustered based on sequence similarity, as detected with the BLAST-Like Alignment Tool (BLAT) (Kent, 2002) (tileSize = 10, stepSize = 5). Sequences with over 90% identity were grouped as a single SLAF locus. Then, the SNP loci of each SLAF locus were detected between parents, and SLAFs with more than three SNPs were initially filtered out. Thereafter, the alleles of each SLAF locus were defined according to the parental reads with a sequencing depth > 10-fold, while for each offspring, the reads with a sequencing depth > 5-fold and integrity > 70% were used to define the alleles. A chi-square test was performed to examine segregation distortion. Markers with significant segregation distortion (*P* < 0.05) were initially excluded. SLAFs with dimorphic SNPs were identified as polymorphic and considered as potential markers. All polymorphic SLAF loci were used to genotype the parents and offspring. The marker coding of the polymorphic SLAFs was analyzed according to the cross-pollinator (CP) population type, and the obtained segregation patterns consisted of five segregation types (ab × cd, ef × eg, hk × hk, lm × ll and nn × np).

### Simple Sequence Repeats Marker Screening and Genotyping

In addition to SLAF-seq SNP markers, we also used SSR markers for genotyping and mapping. Among the 47 SSR primers showing polymorphism between the two parents, 23 genomic SSRs came from the International Consortium of Sugarcane Biotechnology (ICSB) (Cordeiro et al., 2000), and 24 EST-SSRs were designed and developed by the Sugarcane Research Institute of Guangxi Academy of Agricultural Sciences (SRI-GXAAS) (Gao et al., 2020; Supplementary Table 1). Each marker was tested against the expected ratios using the chi-square test. In the genome, only the markers showing a 1:1 ratio (markers present once in one parental genome) was used for mapping. The SSR markers were labeled using the original name followed by a letter that denoted the specific allele in descending molecular weight. DNA amplification and capillary electrophoresis were performed as described earlier (Gao et al., 2020).

### Linkage Map Construction

The modified logarithm of odds (MLOD) scores between markers were calculated to assign markers to the linkage groups (LGs). Markers with MLOD scores < 5 were filtered prior to ordering. Then, the SMOOTH error correction strategy was applied according to the parental contributions of genotypes (Os et al., 2005), and a k-nearest neighbor algorithm was applied to impute the missing genotypes (Huang et al., 2012). To ensure efficient construction of a high-quality high-density map, the newly developed HighMap strategy was utilized to order the SLAF markers in each LG (Liu et al., 2014). Then, skewed markers were added to this map by applying the multipoint method of maximum likelihood (Huang et al., 2012).

Sex-specific maps were constructed using markers that were heterozygous in the female or male parent, while the sex-averaged map was established by integrating the parental maps through the anchor markers (markers that were heterozygous in both parents) (Ooijen, 2011). The updated recombination frequencies were used to integrate these two types of parental maps, which optimized the map order in the next cycle of simulated annealing (Liu et al., 2014). For anchored markers, the map distance was calculated as the average across the two parental distances. The remaining markers that segregated in only one of the parents were placed in the consensus map by interpolation or extrapolation, according to the relative position between the flanking anchor markers in the relevant parental map. Map distances were estimated using the Kosambi mapping function (Kosambi, 1943).

### Quantitative Trait Loci Analysis Using the High-Density Genetic Map

QTL analysis for resistance rating was conducted with MapQTL 6^2^ software using the interval mapping method. Two logarithm of odds (LOD) support intervals were constructed as 95% confidence intervals. The significance of each QTL interval was tested with the likelihood-ratio statistic (LOD). The threshold of the LOD score for significance (*P* = 0.05) was determined using 1,000 permutations. The calculation of the percentage of phenotypic variance explained by each QTL (Expl.%) was performed in MapQTL6 based on the population variance found within the segregating population.

### Comparative Genomics

All mapped SLAF markers were searched against the genomes of *S. spontaneum* (Zhang et al., 2018) and sorghum (Lee et al., 2019) using the nucleotide Basic Local Alignment Search Tool (BLASTN) with an *e*-value cutoff of 1e-10. If a single marker sequence was aligned with multiple targets at different positions, only the top-hit (lowest *e*-value) alignment was retained. Genomic synteny was visualized using Spearman’s correlation (Zar, 2005).

### Bulked Segregant RNA Sequencing Analysis

The RNA samples of the two parents, eight progeny with extremely high resistance, and nine progeny with extremely high susceptibility (Supplementary Table 2) selected from the F_1_ population were extracted from their stalks following the protocol of the TRIzol reagent (Invitrogen Life Technologies Co. Ltd). All RNA samples were extracted approximately 3 days after whip emergence. The quality and quantity of the RNA samples were verified using 1.5% agarose gels and a NanoPhotometer^®^ spectrophotometer (IMPLEN, CA, United States). Two RNA bulks were constructed by pooling the RNA of plants with low or high resistance in equal quantities and sequenced using the Illumina HiSeq 2500 paired-end sequencing platform (Illumina, Inc.; San Diego, CA, United States). The raw reads were trimmed by removing reads that contained adapters, reads that contained poly-N sequences, and low-quality reads using in-house Perl scripts. At the same time, the Q30, GC content and sequence duplication levels of the clean data were calculated. The clean reads were analyzed and mapped to the *S. spontaneum* AP85-441 genome using STAR software (Dobin et al., 2013). The curated data has been submitted to the CNGB sequence Archive (CNSA) of the China National GeneBank DataBase (CNGBdb; accession ID CNP0002008).

For differential gene expression detection, a fold change ≥ 2 and FDR < 0.01 were used as the screening standards. The significance of the difference in the *p*-value obtained from original hypothesis testing with the Benjamini-Hochberg revision method was revised, and the FDR was considered the key indicator for the screening of differentially expressed genes (DEGs).

The Euclidean distance (ED) algorithm (Hill et al., 2013) was used to screen significant SNP markers from the RNA pools to evaluate the regions related to the investigated characteristics. The formula of the ED algorithm was as follows:

$$E\,D=\sqrt{{\left(A_{m\,u\,t}-A_{w\,t}\right)}^{2}+{\left(C_{m\,u\,t}-C_{w\,t}\right)}^{2}+{\left(G_{m\,u\,t}-G_{w\,t}\right)}^{2}+{\left(T_{m\,u\,t}-T_{w\,t}\right)}^{2}}$$

To eliminate false-positive loci, the locations of markers in the genome were used to match the ED values of markers on the same chromosome. According to the correlated threshold value, the region above the threshold value was selected and considered to be related to the target characteristics. The frequency of the alleles of each base pair in the RNA pools was recorded, and the original ED value of each locus was calculated. To eliminate background noise, the 4th power of the calculated original ED value was selected as the associated value and then matched using the sliding window method on the chromosome with a window size of 2 Mb and a step size of 100 kb.

### Co-localization of Quantitative Trait Locis via Genetic Mapping and the Bulked-Segregant RNA Sequencing Method

In the present study, the genetic map was developed without a reference sugarcane genome, whereas the genome of *S. spontaneum* was employed as a reference genome in the BSR-seq analysis. To jointly analyze the localized QTLs identified with both methods, a BLASTN analysis of the SNP markers located in QTLs from a genetic map including the unigenes in the QTLs of the *S. spontaneum* genome from BSR transcripts was performed (identity = 90%). The QTLs containing the identified SNP markers were considered to be co-localized.

### Screening for Candidate Genes

To screen for potential candidate genes, a BLASTN analysis of SNP markers located in QTLs from the genetic map including unigenes from BSR transcripts was performed (identity = 90%). Unigene libraries were constructed with the reference-free genome and the *S. Spontaneum* genome as reference genomes. The identified unigenes were annotated by comparison with public databases, including the Nr, KEGG, Pfam and GO databases.

### Development of Kompetitive Allele-Specific PCR Markers for Smut Resistance Screening

A set of polymorphic SNPs identified in the QTLs strongly associated with smut resistance was selected for KASP marker assay development. To obtain reads longer than SALF-seq, two parents were re-sequenced and the sequences were aligned to *S*. *spontaneum* genome (Zhang et al., 2018) using Burrows-Wheeler Alignment (BWA) Tool (Li and Durbin, 2009). SNPs were called using haplotypecaller of GATK (McKenna et al., 2010). 77 SNPs in the smut resistance QTLs were carefully screened and those with clear fluorescence signal in the two parents according to KASP assay (Semagn et al., 2014) were then further validated by using them to genotype the 90 F_1_ progeny with extreme phenotypes. Based on smut inoculation (phenotypic screening for resistance) data, progeny with scores of 1–4 were classified as “resistant,” and those with scores of 5–9 were grouped as “susceptible.” The tested progeny were considered as “resistant” or “susceptible” for marker assay validation for its diagnostic potential. The *t*-test of each marker was conducted for smut response scores collected annually.

## Results

### Analysis of Phenotypic Data

**FIGURE 1:**
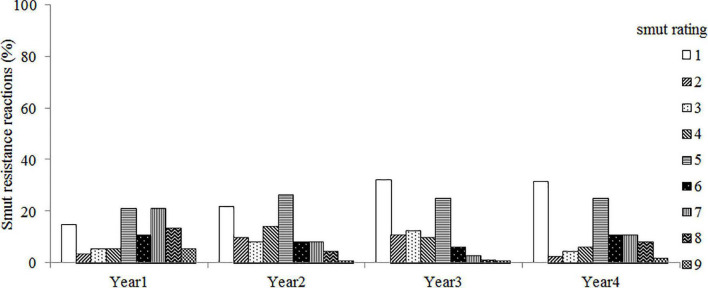
The distribution of smut resistance reaction in sugarcane varieties GT21 and ROC25 (parental clones) and their F_1_ progeny (192 clones, mapping population) across four trials conducted over 4 years (2015–2018). Sugarcane varieties NCo310, NCo376 and F134 were included as control. Clone smut response reaction was graded according to a 1–9 rating scale (1 = highly resistant; 2 and 3 = resistant; 4 = moderately resistant; 5 = moderately susceptible; 6 and 7 = susceptible; 8 and 9 = highly susceptible).

The artificial inoculation showed that the parental line ROC25 was highly resistant to smut disease with an average disease incidence of 1.6%, while 45.3% of GT21 plants, the other parent and a smut susceptible variety, were succumbed to the disease (Supplementary Table 3). The control accessions NCo376 and F134 were susceptible to smut disease, with an average incidence of ∼32%. NCo310 was immune to inoculation, indicating that the physiological race of the isolated strain (*S. scitamineum*) was race 2. The broad-sense heritability (*H*^2^) of smut resistance was 0.87, suggesting a strong genetic control for smut resistance in the test population. However, the distribution of smut resistance reaction in the mapping population over 4 years of trial revealed large environmental influence (*p* <0.001) on smut incidence (Figure 1).

### Identification of Specific Locus Amplified Fragment Markers and Genotyping

High-throughput sequencing of SLAF library yielded 1,881,383,376 high-quality paired-end reads with an average Q30 value of 93.75% and an average GC content of 45.34% (Table 2). A total of 504,537 SLAFs were defined, of which 293,039 were detected in the female parent and 334,654 were found in the male parent. In male and female parents, the total number of SLAF reads were 6,222,889 and 15,086,905 with each SLAF having an average coverage of 21.24- and 45.08-fold, respectively (Table 2). In the progeny population (192 clones), the average number of SLAFs was 195,353, with a coverage of 10.69-fold for each progeny.

**TABLE 2:** SLAF sequencing data summary.

Clone	Raw reads	Q30 (%)	GC content (%)	Number of SLAF reads	Number of total reads	Average sequencing depth
GT21	28,294,864	94.22	44.83	293,039	6,222,889	21.24X
ROC25	61,264,088	92.53	44.21	334,654	15,086,905	45.08X
Average in progeny	9.306,430	93.76	45.38	195,353	2,088,095	10.69X
Total	1,881,383,376	93.75	45.34	504,537	/	/

**FIGURE 2:**
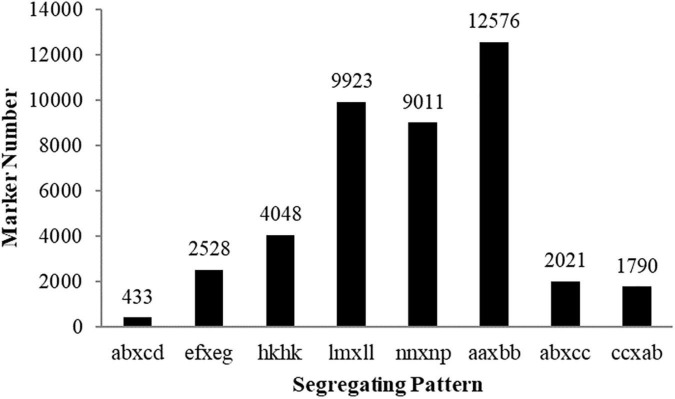
Number of sugarcane SLAF markers in the eight segregation patterns.

Among the 504,537 SLAFs that were defined, 148,500 were polymorphic, with a polymorphism rate of ∼29.0%. Among the polymorphic SLAFs, 42,330 high-quality markers were classified into eight segregation patterns (Figure 2). As shown in Figure 2, 12,576 markers were homozygous in the two parents with a genotype of aa or bb, which belonged to the unsegregated patterns in the progeny. After filtering out these unsegregated markers and low-quality SLAF markers, 8,149 markers conformed to the CP type (Supplementary Table 4). From the 8,149 CP type markers 3,068 with an integrity ≥ 70% and depth ≥ 5 were defined as effective markers and used for subsequent genetic linkage mapping (Supplementary Table 5).

### Construction of Genetic Linkage Maps

After linkage analysis, a high-density genetic map was constructed using the SLAF and SSR markers. The map included 3,088 markers (3,068 SLAF and 20 SSRs) on 60 LGs, spanning 6,066.63 cM, with an average distance of 1.96 cM between adjacent markers (Table 3). A total of 20 SSR markers obtained from 10 primer pairs were distributed in 11 linkage groups, among which the maximum number (4) of SSR markers was included in LG59. On average, 51.47 markers were assigned to each LG, with a length of 101.00 cM (Table 3). The largest LG was LG55, containing 119 markers with a length of 408.19 cM and an average distance of 3.43 cM between adjacent markers. The smallest LG was LG16, containing only 30 markers with a length of 22.50 cM and an average distance of 0.75 cM between adjacent markers (Table 3).

**TABLE 3:** Descriptions of characteristics of 60 linkage groups.

Linkage group	Marker types and numbers			Size (cM)	Average distance (cM)	Largest gap (cM)	Gap ≤ 5 cM (%)
	Total	SNP	SSR				
1	32	32	0	235.59	7.36	28.41	56.25
2	20	20	0	148.09	7.40	58.33	50.00
3	22	22	0	95.85	4.36	53.37	86.36
4	23	23	0	58.03	2.53	11.31	91.30
5	20	20	0	28.58	1.43	9.12	95.00
6	20	20	0	169.28	8.46	22.53	40.00
7	22	22	0	50.79	2.31	5.53	90.91
8	39	39	0	112.33	2.88	23.89	84.62
9	21	21	0	104.92	5.00	19.66	71.43
10	33	33	0	84.55	2.56	19.67	84.85
11	94	93	1	90.38	0.96	17.62	95.74
12	27	27	0	23.70	0.88	4.06	100.00
13	40	40	0	67.43	1.69	9.10	92.50
14	54	52	2	96.45	1.79	18.94	96.30
15	74	74	0	57.91	0.78	12.93	98.65
16	30	30	0	22.50	0.75	2.71	100.00
17	20	20	0	112.61	5.63	47.95	75.00
18	33	32	1	35.19	1.07	4.42	100.00
19	57	57	0	92.87	1.63	21.38	98.25
20	25	24	1	140.9	5.64	56.842	84.00
21	34	34	0	157.35	4.63	21.77	71.43
22	22	22	0	131.86	5.99	20.41	68.18
23	32	32	0	228.59	7.14	53.96	71.88
24	36	36	0	73.03	2.03	7.95	91.67
25	60	60	0	116.73	1.95	10.67	90.00
26	88	88	0	146.61	1.67	21.82	92.05
27	59	59	0	61.14	1.04	6.38	98.31
28	53	53	0	189.35	3.57	28.42	83.02
29	42	42	0	68.03	1.62	7.94	92.86
30	21	21	0	125.28	5.97	28.60	66.67
31	93	92	1	129.49	1.39	9.37	95.70
32	56	56	0	56.03	1.00	6.20	96.43
33	142	139	3	119.28	0.84	10.84	99.30
34	100	99	1	99.24	0.99	30.14	98.00
35	45	45	0	133.34	2.96	28.13	82.22
36	107	107	0	120.14	1.12	19.98	95.33
37	23	23	0	27.96	1.22	4.51	100.00
38	107	107	0	104.75	0.98	5.25	99.07
39	26	26	0	27.79	1.07	5.26	96.15
40	43	43	0	39.08	0.91	3.78	100.00
41	28	28	0	99.88	3.57	36.83	82.14
42	78	78	0	67.01	0.86	4.89	100.00
43	31	31	0	133.18	4.30	24.69	77.42
44	26	26	0	81.37	3.13	33.53	88.46
45	31	31	0	22.53	0.73	4.84	100.00
46	27	27	0	82.20	3.04	17.88	74.07
47	26	26	0	71.13	2.74	16.32	88.46
48	24	24	0	64.66	2.69	8.86	91.67
49	20	20	0	58.08	2.90	10.23	80.00
50	22	22	0	40.26	1.83	8.78	90.91
51	35	33	2	230.65	6.59	50.07	60.00
52	37	35	2	158.39	4.28	35.51	75.68
53	29	29	0	24.95	0.86	5.21	96.55
54	28	28	0	65.85	2.35	20.16	85.71
55	119	119	0	408.19	3.43	48.40	80.67
56	23	23	0	97.02	4.22	39.02	78.26
57	88	86	2	103.46	1.18	12.03	96.59
58	22	22	0	27.23	1.24	6.40	90.90
59	368	364	4	117.34	0.32	11.68	99.73
60	131	131	0	130.23	0.99	8.89	96.95
Maximum	369	364	4	408.19	8.46	58.33	100.00
Minimum	20	20	0	22.50	0.32	2.71	40.00
Total	3,088	3,068	20	6066.63	1.96	/	/
Average	51.47	51.13	0.33	101.11	/	/	86.89

### Analysis of Quantitative Trait Locis

**FIGURE 3:**
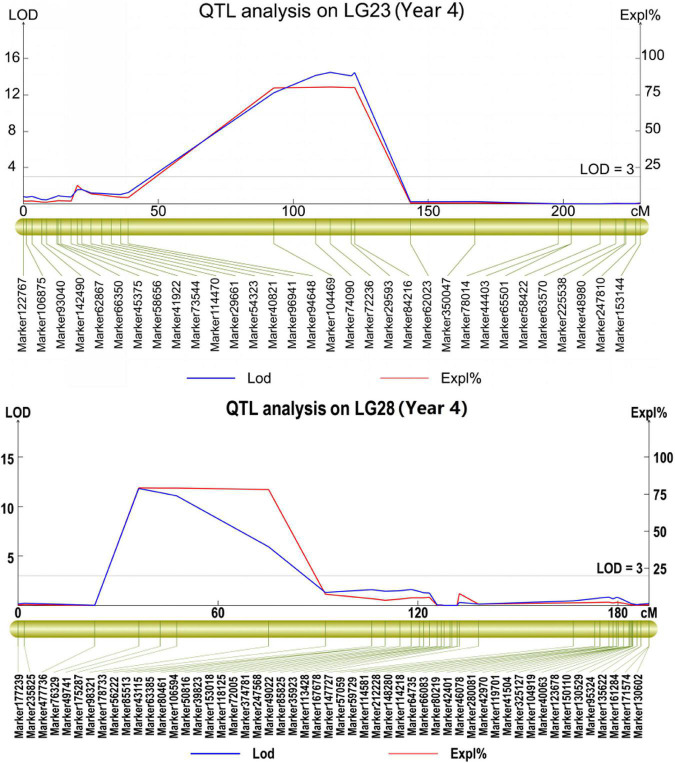
Some repeatable major QTLs associated with smut resistance identified in the mapping population.

The phenotypic data obtained from the 4 years of the study were analyzed separately. As a result, a total of 21 QTLs were mapped, with a phenotypic variance explanation (PVE) of more than 8.0% (Table 4 and Supplementary Table 5). Among these QTLs, 10 repeatable QTLs were identified in at least 2 years (Table 4). Two QTLs identified in 2 years located in LG2 and LG59 explained 77.4∼78.9 and 8.0∼16.8% of the observed phenotypic variance with LOD values of 6.60∼12.72 and 3.16∼4.97, respectively. Among the QTLs identified in 3 years, five QTLs were located in four different chromosomes, on each in LG17, LG23 and LG28 and two in LG1, with PVEs ranging from 60.2 to 80.4% and LODs ranging from 3.03 to 14.48 (Table 4 and Figure 3). The remaining three QTLs were confirmed in all 4 years and were distributed in different LGs (LG20, LG22 and LG51), with PVEs ranging from 58.4 to 81.7% and LODs ranging from 3.27 to 14.70. Further, three QTLs (*qSR20*, *qSR22* and *qSR23*) explained the highest proportion of phenotypic variance (more than 80%) (Table 4 and Figure 3).

**TABLE 4:** Repeatable QTLs associated with the smut resistance of F_1_ population derived from GT21 × ROC25 cross.

Year	QTL	Linkage group	Map position		Marker number	LOD	PVE (%)*^a^*
			Start (cM)	End (cM)			
3/4	*qSR2*	2	41.676/34.008	47.471	2/3	6.60∼12.72	77.4∼78.9
1/4	*qSR59-1*	59	17.219/17.365	18.196/17.462	7/2	3.16∼4.97	8.0∼16.8
1/2/4	*qSR1-1*	1	0	0/0/28.408	1/1/2	3.04∼12.99	60.2∼79.8
1/2/4	*qSR1-2*	1	93.382	124.779	2/3	3.85∼5.30	60.5∼66.9
2/3/4	*qSR17*	17	74.538	74.538	1	3.46∼12.94	65.9∼79.1
2/3/4	*qSR23-1*	23	92.805	122.732	5	3.10∼14.48	62.4∼80.4
2/3/4	*qSR28*	28	42.678/32.216/32.216	42.678/47.615/75.198	1/3/4	3.03∼12.77	66.1∼79.2
1/2/3/4	*qSR20*	20	17.865	21.797/21.797/73.919/73.919	2/2/6/6	3.72∼13.54	58.4∼81.7
1/2/3/4	*qSR22*	22	0	3.75/14.701/14.701/14.701	3/5/5/5	3.27∼14.70	67.4∼80.3
1/2/3/4	*qSR51-1*	51	45.545	49.816/49.816/115.106/49.816	2/2/6/2	3.76∼14.53	63.5∼79.5

*^a^PVE, Phenotypic variance explained.*

### Comparative Genomic Analysis

Comparative genomic analysis was performed between modern sugarcane cultivar LGs and *S. spontaneum* and sorghum chromosomes. Among the 3,068 sequences of SNP markers, a total of 819 (26.7%) markers were mapped to the genome of *S. spontaneum* while 415 (13.5%) markers were mapped to sorghum genome (Supplementary Table 7). Collinearity analysis with Spearman correlation threshold value > 0.8 has identified 16 and six LGs in the genetic map that were shared with the *S. Spontaneum* and sorghum genomes, respectively (Supplementary Table 8). Based on both the number of mapped markers and the identified number of LGs with Spearman correlations > 0.8, sugarcane and *S. spontaneum* exhibited a much closer relationship with each other than the relationship observed between sugarcane and sorghum. Therefore, the subsequent BSR-seq analysis was performed by referring to the *S. spontaneum* genome due to the high degree of synteny and collinearity between the analyzed clones of sugarcane and *S. spontaneum*.

### Bulked-Segregant RNA Sequencing Analysis for Smut Resistance

A total of 22,901193, 21,128245, 67,348551, and 55,829076 clean reads were obtained for the two parents and the two extreme F_1_ pools (Table 5). These clean reads were separately mapped to the *S. spontaneum* AP85-441 genome, and a > 90% mapped rate was obtained for the resistant parent and pool, while an approximately 40% mapped rate was obtained for the susceptible parent and pool (Table 5). Then, the clean reads were mapped to the *S. scitamineum* genome,^3^ and an approximately 50% mapped rate was obtained for the susceptible parent and pool (Table 5). This indicates that the infected stems of the susceptible plants were invaded by the fungus *S. scitamineum*. The following analysis was performed after eliminating the data from *S. scitamineum*.

**TABLE 5:** Sequencing reads for the association analysis using bulked segregant RNA sequencing.

Genotype	Clean reads	Q30 (%)	Mapped reads to *S. spontaneum* (%, mapped radio)	Mapped reads to *S. scitamineum* (%, mapped radio)
ROC25	22,901,193	93.74	21,330,456 (93.14)	4,580 (0.02)
GT21	21,128,245	94.18	8,751,551 (41.42)	11,153,601 (52.79)
High resistant pool	67,348,551	94.71	61,646,969 (91.53)	6,735 (0.01)
High susceptible pool	55,829,076	94.65	26,110,833 (46.76)	27,021,273 (48.40)

A total of 1,251 DEGs were identified between the resistant parent + pool and the susceptible parent + pool, which included 590 up-regulated and 661 down-regulated genes. Between the resistant parent and the resistant pool there were 434 up-regulated and 385 down-regulated genes, whereas between the susceptible parent and the susceptible pool there were 242 up-regulated and 285 down-regulated genes. In total, 1,251 DEGs were annotated based on biological database (Nr, KEGG, Pfam and GO), and among them 32 were shown to be involved in the resistance response, including 6 up-regulated and 26 down-regulated DEGs between the resistant parent + pool and the susceptible parent + pool.

**FIGURE 4:**
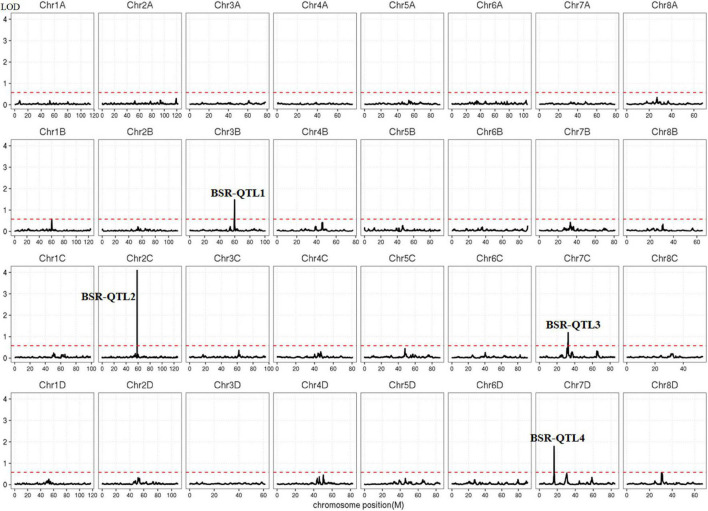
The distribution of QTLs associated with smut resistance in the *Saccharum spontaneum* AP85-441 genome. The dotted line represents the threshold (0.578).

Polymorphic SNPs between the two pools were used to map the loci for smut resistance in the genome of *S. spontaneum*. The threshold value for correlation analysis was set at 99% of the fitted values of all loci, and the calculated threshold value was 0.578 (Figure 4). According to the correlated threshold value, the region above the threshold value was considered to be associated with smut resistance. Finally, four QTLs were identified on chromosomes 2 (BSR-QTL1), 3 (BSR-QTL2) and 7 (BSR-QTL3 and BSR-QTL4) in *S. spontaneum* (Figure 4), with the highest significant peak found for BSR-QTL2. The physical intervals of these four QTLs were 55.6–58.4, 59.3–60.1, 31.0–32.9, and 29.0–31.4 Mb, respectively, with an average length of 1.975 Mb. Through the sequence alignment of the QTLs mapped from the genetic map for modern sugarcane cultivars and the genome of *S. spontaneum*, six markers of the four QTLs from the genetic map were co-localized within BSR-QTL2 and BSR-QTL3 (Table 6).

**TABLE 6:** The co-located QTLs mapped in the genetic map and *Saccharum spontaneum*.

Co-located QTLs		Number of co-located markers
QTL in the genetic map	QTLs by BSR-Seq in *S. spontaneum*	
*qSR28*	BSR-QTL2	1
*qSR59-1*	BSR-QTL2	1
*qSR59-2*	BSR-QTL2	2
*qSR60*	BSR-QTL2, BSR-QTL3	2

### Screening for Candidate Resistant Genes

Using BLASTN, SNP markers located in QTLs from the genetic map were searched against two unigene libraries constructed with or without a reference genome. 64 markers were mapped against the unigene library constructed with the reference genome. However, only one unigene was functionally annotated, encoding a plant hormone signal transduction-related protein (Supplementary Table 9). 63 markers were mapped against the unigene library constructed by *de novo* transcript assembly. After functional annotation, 43 unigenes were mapped to the database (Supplementary Table 9). Four unigenes related to disease resistance were obtained, all of which were located in major QTLs, two in *qSR59-1*, one in *qSR59-2* and one in *qSR20*. Two of these unigenes encoded disease resistance proteins located in the same QTL (*qSR59-1*). The remaining two unigenes were annotated as leucine-rich repeat genes, one of which was located in *qSR20* and was identified in experiments repeated over 3 years.

### Development of Candidate Kompetitive Allele-Specific PCR Markers for Smut Resistance Screening

To develop the diagnostic markers for smut resistance screening, KASP assays were designed for SNPs in the six smut resistance QTL intervals (*qSR2*, *qSR22*, *qSR23-1*, *qSR51-1*, *qSR59-1*, *qSR59-2*). Among the 77 SNPs selected for KASP assays, 49 were successfully genotyped in the two parents, and 24 SNPs were in line with genotypes in our genetic map (Supplementary Table 10). Notably, we found that five markers were highly significantly associated with smut resistance based on *t*-test analysis (*P* ≤ 0.0003–0.01) and one marker association was significant (0.04) (Table 7). The KASP marker A014692 was the most strongly associated one (*P* ≤ 0.0003), which was located in QTL *qSR59-2*.

**TABLE 7:** Sugarcane SNP-based KASP markers associated with smut resistance.

KASP marker	QTL	*P*-value
A014653	*qSR51-1*	0.018357
A014666	*qSR23-1*	0.012546
A014669	*qSR23-1*	0.019141
A014680	*qSR59-2*	0.044136
A014692	*qSR59-2*	0.000387
A014713	*qSR59-2*	0.017852

## Discussion

Plant disease resistance is generally considered a quantitative trait and is governed by multiple genes. Previous studies revealed that the sugarcane smut resistance phenotype shows continuous variation, indicating that the trait is a quantitative and controlled by multiple genes (Chao, 1991). In the present study, the smut resistance reactions of the mapping population also showed continuous variation, and we obtained a total of 25 major QTLs by combining QTL mapping using a high-density genetic map and BSR-seq. Among these QTLs, 21 were located in the genetic map of modern sugarcane cultivars, four were located in the genome of *S. spontaneum*, and four were co-localized in more than one genome. However, the highly resistant control variety NCo376 showed susceptibility, which has also been found in other studies (Liu et al., 2011; Gao et al., 2013; Xiong et al., 2013). The potential reasons for this susceptibility include variety degeneration (NCo376 is a very old variety), evolution of a new physiological race of smut pathogen (Shen and Deng, 2011; Liu et al., 2018) and the strong environment and genotype × environment interaction effects on smut expression (Hoy and Grisham, 1988; Chao et al., 1990). Furthermore, a single physiological race was selected for artificial inoculation in this study, the aim of which was to eliminate the mutual influence of resistance or susceptibility genes in different physiological races under mixed inoculation, which could cause inaccurate QTL identification.

A previous study utilized populations generated from the same male parental line (ROC25) for linkage map construction (Liu et al., 2010). However, only 133 traditional molecular markers (AFLPs and SSRs) were mapped in 266 progeny. The present study mapped many more markers (3,068 SNPs and 20 SSRs) than have been mapped in previous studies, spanning a distance of 6,066.63 cM with a density of 1.96 cM/SNP. Among the mapped SSR markers, mSSCIR17 and mSSCIR36, were mapped in both linkage maps of ROC25. High-throughput next-generation sequencing (NGS) technologies are now providing new opportunities for discovering molecular markers, especially SNPs, at the genome-wide level (Davey et al., 2011). Some of these techniques, such as genotyping-by-sequencing (GBS) (Balsalobre et al., 2017; Yang et al., 2018) and SNP array analysis (You et al., 2019), have been used to construct genetic maps and for QTL mapping in sugarcane, indicating their power and effectiveness in sugarcane high-throughput genotyping. In the present study, we first combined the SNP markers developed on the basis of SLAF-seq and SSR markers to construct a high-density genetic map with an average distance of 1.96 cM between adjacent markers, which showed, for the first time, that SLAFs can serve as a valuable additional tool for sugarcane genetic studies. Thus far, all the maps constructed using NGS technologies remain unsaturated due to the complexity of the sugarcane genome and the absence of a statistical genetic model.

It has been demonstrated that combining QTL mapping using a high-density genetic map and BSR-seq is a powerful and cost-effective approach for decoding the genetic architecture underlying traits-of-interest (Liu et al., 2016). In the present study, we used bulk segregant RNA-seq for exploring the genes (and genetic loci) involved in sugarcane smut resistance. Based on the SNPs generated from two parents and two RNA pools from plants with extreme but contrasting smut resistance, four significant peaks were observed on chromosomes 2 (BSR-QTL1), 3 (BSR-QTL2) and 7 (BSR-QTL3 and BSR-QTL4). Through the sequence alignment of QTLs from the genetic map of modern sugarcane cultivars against those from the genome of *S. spontaneum*, six markers of the four QTLs from the genetic map were co-localized within BSR-QTL2 and BSR-QTL3. Among these markers, only one marker each in *qSR28* and *qSR59-1* was included in BSR-QTL2, possibly because of the insufficient marker density in *qSR28* and *qSR59-1*. Furthermore, the two markers in *qSR60* were included in BSR-QTL2 and BSR-QTL3, indicating that BSR-QTL2 and BSR-QTL3 showed potential homology. As the next step, we aim to develop new markers in the coding regions on both sides of the co-localized markers in the *S. spontaneum* genome, especially for those in *qSR28* and *qSR59-1*, which were identified repeatedly. Also, it is important to note that, in this study four markers directly related to smut resistance were obtained through the sequence alignment of SNP markers located in QTLs from a genetic map against a unigene library constructed without a reference genome.

A gene may be localized in a species with an unsequenced genome by using a genetic map or sequencing information from a related species with a known genome through comparative genomics according to the collinearity of the related species’ genome. This approach can reveal the potential functions of genes and the internal structure of the genome in species with unsequenced genomes. By applying comparative genomics analysis, collinear genomic regions of the wheat powdery mildew resistance gene (*MlHLT*) were identified in *Brachypodium distachyon*, rice and sorghum, and three new polymorphic markers were developed (Wang et al., 2015). In sugarcane, comparative mapping with sorghum and rice to saturate markers in the target area of the sugarcane brown rust resistant gene *Bru*1 was described by Asnaghi et al. (2000) and Cunff et al. (2008). Their results demonstrated that sugarcane and sorghum genomes are mostly collinear in genic regions (Asnaghi et al., 2000; Cunff et al., 2008). The complete sequence of *S. spontaneum* genome now being available (Zhang et al., 2018), we compared the collinearity between the genetic map of sugarcane and the genomes of sorghum and *S. spontaneum* in this study, and found that the relationship (collinearity) between sugarcane (*Saccharum* spp.) and *S. spontaneum* was much closer than that between sugarcane and sorghum. Furthermore, six markers were co-localized through the sequence alignment between the QTLs from the genetic map without a reference genome and BSR transcripts with the *S. spontaneum* genome as a reference. Therefore, we support the *S. spontaneum* genome as an additional useful option for sugarcane reference-based NGS sequence analysis. Nevertheless, genetic and molecular breeding studies in sugarcane remain very difficult due to the complex genome structure and genome behavior of modern sugarcane hybrids. Hence, a reference genome of modern sugarcane hybrids is needed for comprehensive molecular genetic studies.

Marker-assisted selection for sugarcane smut resistance has shown limited progress. Although some markers linked to sugarcane smut resistance have been obtained (Xu and Chen, 2004; Wei et al., 2006; Gao et al., 2013; Esh and Nasr, 2014; Khan et al., 2017), there have been no reports of sugarcane smut resistance MAS using the above markers, possibly because of the low degree of linkage between markers and resistance genes. In this context it is worth noting that, one of the previous sugarcane marker-trait association studies using a panel of 154 clones derived from diverse pedigree reported SSR and AFLP markers associated with resistance to smut, pachymetra root rot, Fiji leaf gall and leaf scald diseases (Wei et al., 2006). This study, while providing promising results with potential for MAS in sugarcane, showed that a significant proportion of marker-trait association detected was due to the effects of embedded population structure of test clones and random effects, and not due to the true physical linkage between marker and the genetic locus conferring disease resistance. This illustrates the disadvantage of using populations produced from multiple parents to identify robust marker-trait association as opposed to using a bi-parental population as deployed in our study. Thus, the single-cross population from parents with contrasting smut resistance phenotype combined with the power of SLAF-seq and BSR-seq enabled us to identify QTLs and markers strongly and stably linked to smut resistance in this study. Among them six SNP markers were used to develop practically useful KASP marker assay, validated in a test population, for smut screening. This is the first report of KASP assay for smut resistance screening in sugarcane. KASP assays based on tightly linked markers for routine disease resistance screening have been developed for different plant diseases in other crops (Cao et al., 2020; Chen et al., 2021).

The genome of modern sugarcane cultivars is highly polyploid (12x), aneuploid, of interspecific origin, and contains 10 Gb of genomic DNA (D’Hont, 2005), resulting in a large distance between markers and relevant genes, thus influencing the precision and stability of linked markers. On the other hand, for highly polygenic quantitative trait genes, effective MAS may be possible only when all major QTLs have been identified and the complex hereditary basis has been resolved into independent Mendelian factors. For sugarcane brown rust resistance, Le Cunff et al. (2008) used comparative genomics strategies to isolate the *Bru1* gene by chromosome walking and finally developed a high-resolution map including markers at 0.28 and 0.14 cM on either side of the gene and identified 13 markers co-segregating with *Bru*1. Genomic and genetic studies of onion are equally difficult due to its large genome size (16.3 Gb), and the lack of reference genome (Khosa et al., 2016). Hence, Bang et al. (2013) performed genome walking to obtain the flanking sequences linked to the Ms locus controlling fertility restoration to develop a co-dominant marker in onion. In the present study, we obtained a total of 10 repeatable QTLs (PVE > 8.0%) for smut resistance from the GT21 × ROC25 segregating population using a high-density genetic map. As with the examples noted above, exploring comparative genomics strategies employing *S. spontaneum*, sorghum, rice and maize as model species would greatly increase the density of the genetic and physical maps of these major QTL regions, which will help perform chromosome walking to identify target genes and develop more closely linked markers in the future.

## Conclusion

Resistant varieties remain the most effective and economic solution to manage sugarcane smut disease. However, smut resistance is a moderately heritable trait with strong genotype x environment interaction. This limitation, along with the undesirable and difficult-to-break trait linkages in sugarcane make breeding for highly productive smut resistant varieties challenging. Marker-assisted selection could be an effective strategy for disease resistance screening in breeding programs. In this study, we first proved that combining QTL mapping using a high-density genetic map and BSR-seq is a powerful approach for the molecular mapping of underlying traits of interest in sugarcane. This was validated with the identification of QTLs for smut resistance, especially the co-localized QTLs, candidate genes and SNP markers related to resistance. The KASP assay developed based on SNP markers closely linked to smut disease resistance holds promise for developing practically useful high-throughput PCR assays for smut resistance screening in sugarcane breeding populations.

## Data Availability Statement

The datasets presented in this study can be found in online repositories. The names of the repository/repositories and accession number(s) can be found in the article/Supplementary Material.

## Author Contributions

YG and GZ: conceptualization. YG, JL, SZ, YH, and BZ: methodology. YX, RY, CY, and HZ: formal analysis and investigation. YG: writing—original draft preparation. YX and PL: writing—review revision and editing. YG, HT, DH, WH, and WD: funding acquisition. CY and WD: resources and supervision. All authors contributed to the article and approved the submitted version.

## Conflict of Interest

YX was employed by Adsen Biotechnology Co., Ltd. (Urumchi, China). The remaining authors declare that the research was conducted in the absence of any commercial or financial relationships that could be construed as a potential conflict of interest.

## Publisher’s Note

All claims expressed in this article are solely those of the authors and do not necessarily represent those of their affiliated organizations, or those of the publisher, the editors and the reviewers. Any product that may be evaluated in this article, or claim that may be made by its manufacturer, is not guaranteed or endorsed by the publisher.

## Footnotes

1
http://sugarcane.org/sugarcaneproducts/ethanol

2
https://www.kyazma.nl/index.php/MapQTL/

3
https://www.ncbi.nlm.nih.gov/assembly/GCA_001010845.1/
